# One-Way Self-Expanding Growing Rod: A Monocentric Study on 20 Patients

**DOI:** 10.7759/cureus.112560

**Published:** 2026-07-13

**Authors:** Victor Font, Ophea Roy, Laurine Gollentz, Olivier Hild, Yann Chaussy, Jeremie Nallet

**Affiliations:** 1 Pediatric Surgery, Hopital Universitaire Montpellier, Montpellier, FRA; 2 Orthopaedic Surgery, Centre Hospitalier Universitaire de Besançon, Besancon, FRA; 3 Pediatric Surgery, Centre Hospitalier Universitaire de Besançon, Besançon, FRA; 4 SINERGIES (Soins Intégrés, Nanomédecine, IA et Ingénierie pour la Santé), Université Marie et Louis Pasteur, Besançon, FRA; 5 Orthopedic Surgery, Centre Hospitalier Universitaire de Besançon, Besancon, FRA; 6 SINERGIES (Soins Intégrés, Nanomédecine, IA et Ingénierie pour la Santé), Université Marie et Louis Pasteur, Besancon, FRA

**Keywords:** complex spine deformity, early-onset scoliosis, growing rod, new technology in spine surgery, pediatric spine surgery

## Abstract

Introduction

Severe and progressive early-onset scoliosis in children can have serious repercussions on lung development and significantly reduce life expectancy. They present a major challenge to the surgeon, as growth must be preserved. Traditional growing rods and the need for repeated lengthening surgeries are associated with numerous complications and high psycho-socio-economic costs for the patient and family. This study aimed to evaluate the lengthening efficacy of the one-way self-expanding rod (OWSER).

Methods

This retrospective and prospective, monocentric, observational clinical study evaluates the efficacy of lengthening of the self-expanding rod on the growth of 20 children between 2018 and 2023. Questionnaires on quality of life (EOSQ24 or Early Onset Scoliosis Questionnaire), clinical and radiological data were collected preoperatively, then at six weeks, three months, and six months postoperatively, and then annually until the end of the child's growth. The primary endpoint was system and spinal lengthening. Secondary endpoints were maintenance of deformity (Cobb angle), assessment of satisfaction questionnaires (EOSQ24), and complications with revision surgery.

Results

The mean age of patients was 11.5 years, with a mean follow-up time of 32 months. Sixty-five percent (65%) had scoliosis of neuromuscular origin, and 25% of syndromic origin. The T1-T12 segment lengthened by an average of 46 mm between preop and last follow-up and 14.4mm between postop and last follow-up. The T1-S1 height averaged 302 mm preoperatively, 352.9 mm postoperatively, and 385 mm at the last follow-up, a significant increase of 83 mm between preop and last follow-up (p < 0.0001) and a significant increase of 32.1 mm between postop height and the last follow-up (p<0.05). A significant decrease in the mean Cobb angle of 25° was measured between preop and last follow-up. Fifty-five percent (55%) of cases required revision surgery.

Conclusion

This study shows a significant lengthening of the OWSER in 20 patients, as well as satisfactory maintenance of coronal curvature. A longer follow-up period is required to assess long-term evolution.

## Introduction

Scoliosis is a three-dimensional deformity of the spine that, when severe and early in onset, can have implications for lung development and life expectancy.

Spinal deformity in young children is classified into four subgroups [1]: congenital, neuromuscular, syndromic, and idiopathic. Orthopedic treatment with a brace or casting has been validated for early-onset scoliosis, with surgery being required when the deformity is no longer controlled. In growing children, each of these techniques seeks to prevent progression of the deformity and offers correction where possible, whilst permitting the spine to continue growing. These growth-permitting systems fall into three distinct categories, depending on the correction strategy or the force exerted by the implants on the spine, namely, compression, guided growth, or distraction [2].

In compression techniques, a compressive force is exerted on the growth plate of the convexity, thus inhibiting growth through the Hueter-Volkmann principle. Examples of this technique include vertebral body stapling (VBS) [3] and vertebral body tethering (VBT) [4].

In growth-guided techniques, the proximal and distal vertebrae are instrumented, and the spine is allowed to slide along the rod as it grows. Examples of this technique include the modified Luque Trolley [5] and Shilla techniques [6].

Distraction-based techniques involve the application of a longitudinal force at the apex of the deformity. Traditional growing rods (TGR) were first described by Harrington in 1962 and are now the gold standard growth-friendly treatment. Initially, this technique consisted of the placement of a single subperiosteal distraction rod. This has since been extensively modified, and today, it consists of a double rod with fixation points at each end and central connectors to allow lengthening through growth. Unfortunately, these repeated lengthening procedures cause complications in more than one in two patients [7], with a negative impact on the psychological well-being and quality of life of the child and their family [8-10]. To avoid this, magnetically controlled growth rods (MCGR), the Shilla technique, and one-way self-expanding rod (OWSER) have been introduced, which replicate this technique while avoiding repeated planned surgical procedures.

The principle of OWSER is that the domino slides along the notched rod according to the patient's growth. Indeed, two sizes of growth reserve are available: 50 or 80 mm. These sizes are then the potential growth for the patient, starting from immediately postoperative and during the treatment.

As the spine grows, the domino is automatically distracted on the notched rod. Then, a consumption of this growth reserve is the sign of spine growth and device functionality.

Whilst the MCGR and Shilla techniques are established technologies for managing spinal deformity in growing children, the results of OWSER have been limited to a group of patients with neuromuscular scoliosis over an 18-month follow-up [11]. The aim of this study is therefore to evaluate spinal growth by lengthening the system, as well as to describe the complications and maintenance of spinal curvature.

## Materials and methods

We conducted an observational study, with retrospective and prospective inclusion and a prospective follow-up of patients treated surgically for scoliosis between 2018 and 2022 at a university hospital in a pediatric surgery department with OWSER.

Patients were included if they showed a progressive scoliosis with a Risser grade of 0 and a Cobb angle above 40°. Patients were excluded if they endorsed a history of spinal surgery.

The study was conducted after obtaining ethical approval from the Institutional Review Committee (CPP). Written consent was obtained from the parents or guardians of each patient to participate.

Clinical and radiological data were collected preoperatively, then at six weeks, three months, and six months postoperatively, and then annually until the end of the child's growth.

**Figure 1:**
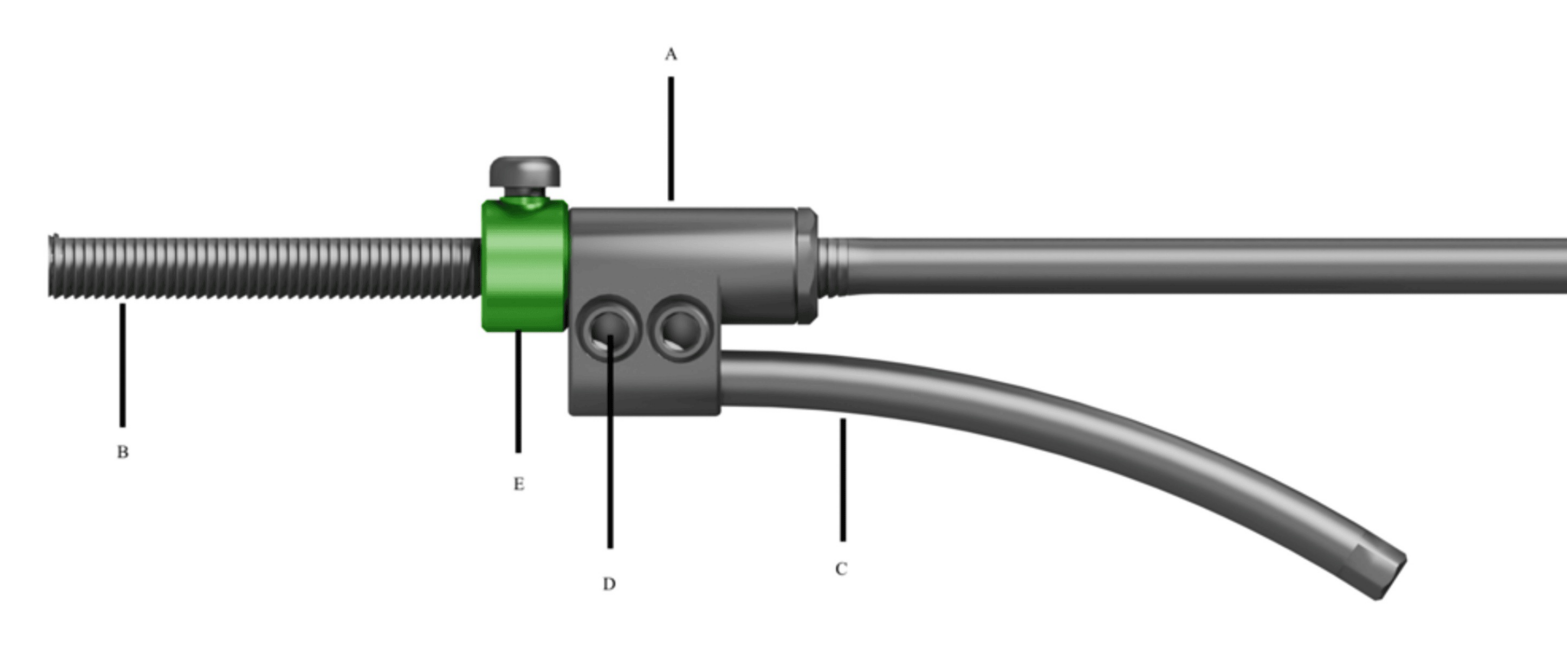
One-way self-expanding growing rod A: Domino body - the domino allows two longitudinal and parallel rods, anchored to the vertebrae and/or pelvis, to be connected, using different anchoring methods (hooks, pedicle screws, illio-sacral screws). B: Notched rod - with a growth reserve of 50 or 80 mm. A physical stop at the end of the rod prevents the rod from going out of the domino when the mechanism reaches the end of its elongation. C: Smooth rod - this stem connects the two poles of the assembly between the thoracic and lumbar or pelvis parts. D: Locking screws - to lock the smooth stem. E: Safety clip - to prevent the notched rod from sliding freely during intraoperative manipulation.

Three fellowship-trained pediatric orthopaedic surgeons performed the surgical procedure between 2018 and 2022. It consisted of a minimally invasive bipolar construct connected by the one-way self-expanding rod (Figure 1). Proximal anchoring was achieved by a pediculo-laminar clamp system; distal anchoring depended on the patient's ambulatory status and may include pelvic fixation if wheelchair bound or pedicle screw fixation of the lumbar spine for ambulating patients. The type of pelvic fixation was based on the surgeon's preference, either with ilio-sacral or sacral alar iliac screws. The self-expanding system came in two forms, one 50 mm and the other 80 mm, depending on the child's estimated growth reserve. A double 5.5 mm titanium rod connected the two anchors.

Clinical and radiological data were collected by a department physician and verified by a clinical research assistant. Preoperative clinical data included age, weight, height, medical and surgical history, and the etiology of the scoliosis (neuromuscular, congenital, or syndromic). Similarly, we recorded any conservative treatments performed preoperatively.

Preoperative radiological measurements were taken using EOS imaging of the whole spine, front and side, whenever possible, or by a full-spine radiograph if not available. Data measured included the coronal angular deformity, T1-T12, and T1-S1 height.

Intraoperative data collected included surgical time, any neurological changes observed on intraoperative monitoring (motor-evoked potentials), blood loss, and instrumented levels with the nature of the instrumentation used (hooks, pedicle screws).

Radiological measurements were collected at each visit: immediate postop, at six weeks, at three and six months, and annually. These included lengthening of the self-expanding system, lengthening of T1-T12 and T1-S1 heights, and various angles for monitoring maintenance of coronal deformity correction (scoliosis, kyphosis, lordosis, and pelvic obliquity).

When the child could not stand in the EOS device, and therefore a full spine radiograph was performed, a measurement calibration method was used.

Early Onset Scoliosis Questionnaire (EOSQ)-24 quality-of-life questionnaires [12] were completed by patients or their parents pre- and postoperatively. All complications and their treatment were collected, whether or not they were related to the self-expanding system.

Qualitative parameters were summarized by their absolute frequency (n) and relative frequency (%). Quantitative parameters were summarized by number of observations, mean, standard deviation, median, quartiles, minimum, and maximum. Nominal categorical variables were compared using the Chi-square or Fisher's exact test. Ordinal qualitative variables were compared using the Wilcoxon test. Quantitative variables were compared by the student's t-test for paired samples or, if the conditions for applying this test were not met, by a Wilcoxon test. Statistical tests were two-tailed. The significance level was set at 5%. Implant survival was calculated by the Kaplan-Meier method with a confidence interval of 95%. The probability of implant survival was calculated by defining failure as return to the theater for all mechanical causes as the endpoint.

## Results

Twenty-two (22) patients were recruited. Two patients, one with a history of spinal surgery and the other lost to follow-up, were excluded. Twenty patients were therefore included.

**Figure 2:**
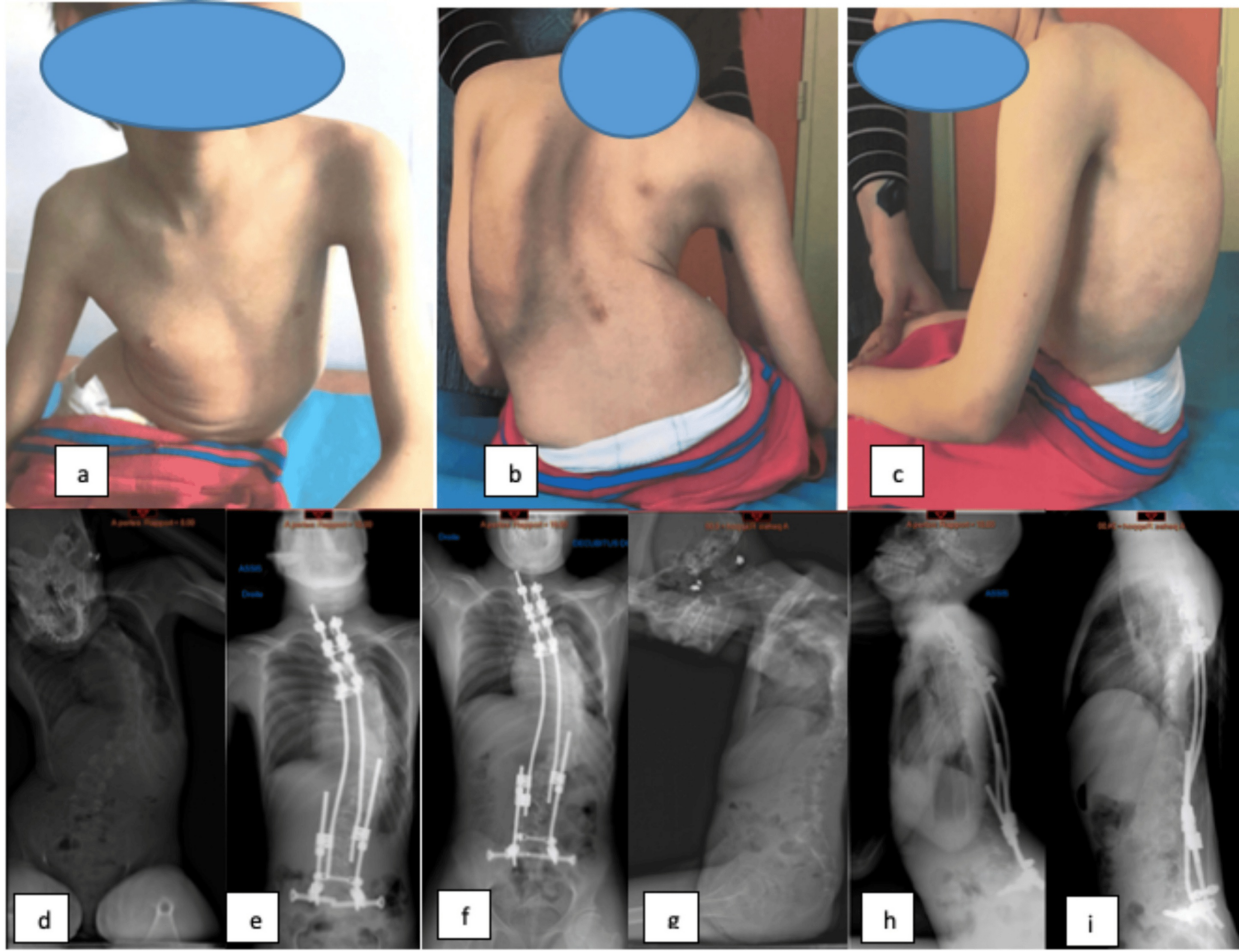
Clinical pictures Example of a patient with cerebral palsy: preoperative clinical aspect from the front (a), back (b), and side (c); radiographs from the front preoperatively (d), immediately postoperatively (e), and four years later (f); and from the side preoperatively (g), immediately postoperatively (h), and four years later (i)

In these patients followed between 2018 and 2023, early spinal deformities were mainly of neuromuscular (65%, i.e., 13 patients) and syndromic (25%, i.e., 5 patients) origin (Figure 2).

 The main characteristics of the patients are described in Table 1.

**Table 1:** Patient characteristics N/A: not applicable

Variable	Mean / n (%)	Min–Max
Age (years)	11	8–15
Weight (kg)	35	19–62
Surgical time (min)	237	174–372
Blood loss (mL)	274	100–700
Hospital stay (days)	11	6–29
Boys	55%	N/A
Girls	45%	N/A
Walker	40%	N/A
Non-walker	60%	N/A
Risser 0	100%	N/A

Of all the patients recruited, 11 were boys (55%) and 9 girls (45%). The average age at the time of instrumentation was 11.5 years (8.3-15.3 years). Average follow-up time was 32 months (7-54 months). Sixty percent (60%) of patients were non-walkers. Hundred percent (100%) of patients had pubertal status classified as Risser 0.

Brace treatment was performed preoperatively in 16 patients (80%) and halo-gravity traction in 3 patients (15%). The surgical procedure consisted of a bipolar set-up. Proximal anchoring began at T1 in 13 cases (65%) and at T2 in 7 cases (35%). Hook fixation was used in 18 patients (90%) and screw fixation in 2 (10%).

Distal anchoring was performed according to the patient's ambulatory status. Of the 14 patients for whom pelvic fixation was performed, eight had sacroiliac fixation, and six had ilio-sacral fixation.

The level of the OWSER was located at the proximal part of the distal incision, between T12 and L4, under the muscle in all cases. Fifty-two point five percent (52.5%; 21/40) were placed at the level of L2-L3. We installed 26 OWSERs with a 50 mm threaded rod reserve and 14 with an 80 mm threaded rod reserve. 

Table 2 and Table 3 show the mean angular deformity, 64° (standard deviation 19) preoperatively, and 33° (standard deviation 13) immediately postoperatively. This shows a correction in curvature of 48%. Moreover, at 4 years, the mean curvature was 39°, i.e., a correction of over 39% compared with the preoperative curvature. The mean Cobb angle was significantly reduced by 25° between preop and follow-up (p < 0.0001).

**Table 2:** Radiographic findings preop, immediate postop, and at last follow-up nd: not disclosed; FU: follow-up; *p<0.05 (vs immediate postop)

Patient		Growth reserve left (mm)			Growth reserve right (mm)			Coronal Cobb angle (°)				Pelvic obliquity (°)		
-		Immediate postop	Last FU		Immediate postop	Last FU		Preop	Immediate postop	Last FU		Preop	Immediate postop	Last FU
1		50	36		50	36		57	29	41		3	3	2
2		80	53		50	10		117	48	60		3	0	2
3		50	39		48	29		80	43	70		25	14	14
4		38	17		38	7		40	29	40		11	8	20
5		80	49		80	31		43	19	38		0	0	0
6		50	26		50	20		50	22	30		0	0	0
7		50	24		50	4		66	25	25		nd	nd	2
8		80	77		80	74		68	26	52		0	6	9
9		50	14		50	14		65	37	36		20	5	10
10		46	-5		51.7	0		75	45	48		24	23	25
11		41	27		50	32		60	28	30		nd	10	6
12		50	48		48	33		90	53	53		0	0	0
13		44	31		55	40		62	56	62		0	2	2
14		43	23		43	24		50	22	24		18	4	9
15		66	17		83	33		55	14	16		6	1	0
16		78	51		80	58.5		90	40	55		23	2	5
17		76	48		75	48		43	20	14		6	0	0
18		46	28		50	32.9		50	20	20		13	5	10
19		73	72		73	70		52	38	40		21	0	2
20		44	24		43	15		67	40	33		14	2	2
Mean (standard deviation)		57 (15)	35 (21)*		57 (15)	31 (20)*		64 (19)	33 (12)	39 (16)*		10.4 (9.5)	4 (2.9)	6.6 (8.2)*

**Table 3:** Radiographic findings preop, immediate postop, and at last follow-up nd: not disclosed; FU: follow-up; *p<0.05 (vs immediate postop)

Patient		T1-T12 height (mm)				T1-S1 height (mm)						Success	
-		Preop	Immediate postop	Last FU	Annual growth (mm/y)	Preop		Immediate postop	Last FU	Annual growth (mm/y)		Defined at least 10 mm/y	
1		196	214	214	0	339		318	355	11		Yes	
2		135	214	219	1	230		305	347	11		Yes	
3		185	194	211	8	225		267	285	8		No	
4		nd	221	239	4	nd		378	410	7		No	
5		200	237	268	12	318		366	430	25		Yes	
6		164	188	214	13	277		288	369	40		Yes	
7		nd	220	220	0	nd		420	420	0		No	
8		159	183	191	2	266		326	353	7		No	
9		nd	219	252	17	nd		377	405	15		Yes	
10		nd	256	257	0	nd		395	417	10		Yes	
11		nd	263	284	8	nd		423	470	18		Yes	
12		203	264	284	9	364		445	475	13		Yes	
13		210	249	275	6	357		417	440	5		No	
14		199	214	218	1	325		370	392	7		No	
15		163	206	214	3	297		358	409	20		Yes	
16		nd	193	194	0	nd		315	352	9		No	
17		nd	198	220	13	nd		317	347	17		Yes	
18		230	233	247	8	350		389	411	13		Yes	
19		nd	132	150	27	nd		251	226	-38		No	
20		163	192	205	22	276		333	365	55		Yes	
Mean (standard deviation)		184 (27)	215 (32)	230 (31)*	8 (8)	302 (48)		353 (54)	385 (62)*	13 (17)		12/20	

These tables summarize the radiographic data measured preoperatively, immediately postoperatively, and at last follow-up. Left and right OWSER reserve consumption is significantly greater at the last follow-up compared with immediate postop, at 23 and 27 mm, respectively (p<0.0001).

T1-T12 height averaged 184 mm preoperatively, 214.5 mm postoperatively, and 230 mm at last follow-up, a significant increase of 46 mm between preop and last follow-up (p=0.002) and a significant increase of 14.5mm between postoperatively and the last follow-up (p<0.05). T1-S1 height averaged 302mm preoperatively, 352.9mm postoperatively, and 385 mm at last follow-up, a significant increase of 83 mm between preop and last follow-up (p < 0.0001) and a significant increase of 32.1 mm between postop height and the last follow-up (p<0.05).

The complications (Table 4) found in this study were mechanical in origin, either due to obstruction of the threaded rod, inducing a crankshaft effect, or migration of the smooth rod due to loosening of the screws of the OWSER. These mechanical complications occurred in 10 patients. Seven of these patients underwent revision surgery for mechanical complications.

**Table 4:** Patient complications

Patient number	Complications	Details of treatment complications	Follow-up (months)
2	Anemia; the threaded rod growing stopped	Transfusion distraction on the threaded rod	45.3
3	Prominent implant; the threaded rod stopped growing	Rod section	27.1
4	Rod fracture	Posterior spine fusion T2-L3	53.8
5	The threaded rod growing stopped	Distraction on the threaded rod	30.9
8	The threaded rod growing stopped	Distraction on the smooth rod because of the OWSER being blocked	44.2
9	Deep infection; migration of the smooth rod	Wound irrigation, antibiotic therapy, VAC therapy (9 revisions), revision with exchange of the connector rod	23
10	Loosening diabolo screws (2)	Mechanical distraction with tightening	27
11	Neurologic: dural breach	Breach closing	31.9
13	Loosening diabolo screws (2)	Mechanical distraction with tightening, revision with OWSER exchange of 80 mm	51
16	The threaded rod stopped growing	-	48.2

In cases where the threaded rod was blocked, we performed one distraction on the smooth rod (because the threaded rod was blocked) and two distractions on the threaded rod. In cases of migration on the smooth rod, we performed a connector stem change in one patient and two mechanical lengthenings with tightening of the screws. We performed the final arthrodesis for a rod fracture.

In all, 11 patients underwent revision surgery (Table 4). Only one patient required multiple revisions in the operating room for wound lavage and treatment with negative pressure therapy. No metallosis was observed in this cohort.

The cumulative survival rate of the implant was estimated to 48% at 4 years (95% CI (24-93%)), taking all returns to theater for all mechanic cause as the endpoint.

The EOSQ-24 score was collected preoperatively and postoperatively in over two-thirds of patients (Table 3). The mean overall preoperative score was 59/100 (standard deviation 30), and the mean overall postoperative score was 63/100 (standard deviation 29).

There was a significant difference for general health, rising from 53/100 preoperatively to 67/100 postoperatively. Satisfaction was also significantly improved from 50/100 preoperatively to 75/100 postoperatively.

## Discussion

This study shows that treatment with a one-way self-expanding rod in patients with scoliosis enables the elongation of the system and growth of the spine while maintaining acceptable curve correction. Mean device growth (right and left) was 25 mm over the follow-up period, with mean elongation of the T1-T12 segment of 46 mm between preop and last follow-up and 83 mm for the T1-S1 segment. The T1-T12 segment growth was 14.5 mm between the immediate postop and the last follow-up, and 32.9 mm for the T1-S1 segment. The scoliosis showed 50% of correction between preoperative and immediate postop, and 39% of scoliosis correction at the last follow-up. Our complication rate was 55%.

Chen et al. published their experiences with 40 children treated with a TGR without fusion and sequential distraction [13]. At the time of final fusion, coronal deformity was improved by an average of 24°, and the measured growth of the instrumented but unfused segments averaged 40 mm, over a mean treatment period of 4.7 years. These results show that our study is comparable with this study.

Marquez-Lara et al. published their results on the maintenance of deformity and increase in thoracic height in a cohort of 24 patients [14]. In fact, at five years, magnetically controlled rods enabled a scoliosis reduction of 25° to be maintained, as well as a mean T1-S1 growth of 102mm. The authors report that 54% of patients required revision surgery over a 2-year follow-up period, which is similar to our cohort, with a mean complication rate of 55%.

In their systematic review, Thakar et al. analyzed the outcomes of a magnetically controlled growing rod in 335 patients. They reported a mean complication rate of 44.5% with an unplanned revision rate of 33% [15]. The same rate of complication (43.9%) was found by Mainard et al. in a nationwide French study with a cohort of 90 patients [16]. This is slightly less than in our cohort, but the concern of metallosis [17] and the need for removal at two years should be considered when choosing this type of growing rod. In our cohort, we did not report significant metallosis.

Similarly, El-Hawary et al. reported that, at the two-year follow-up, vertical expandable prosthetic titanium rib (VEPTR) proved effective in the treatment of infantile idiopathic scoliosis (IIS) without rib anomalies. The T1-S1 length was 27.1 ± 6 cm at immediate postop, and 31.9 ± 5 cm at the 2-year follow-up, which corresponds to a growth of 48 mm vs 83 mm in our study. The scoliosis showed a significant decrease from 72 ± 18° preoperatively to 47 ± 17° immediately postoperatively, and a discreet increase to 57 ± 18° at 2 years. This corresponds to a decrease of 15° of the Cobb angle at the last follow-up vs 20° in our cohort [18]. They also report a complication rate of 49% vs the 55% for our study.

Bess et al. reported the complication rate of TGR techniques in the Growing Spine Study Group of 140 patients who underwent 897 growing rod procedures. In their study, 58% of patients had at least one complication [7]. In our study, 55% of cases required revision surgery, of which 35% for a so-called mechanical complication. We believe it is important to carry out a precise analysis of these cases of failure. Cases of migration on the smooth rod are probably linked to loosening of the locking eccentric screw, for which the laboratory (EUROS S.A., La Ciotat, France) is currently working on modifications. Cases of mechanical blockage of the threaded rod in the device could be explained by the presence of an anti-rotation flat, which, under stress, could block the passage of this threaded rod.

Pawelek et al. published the results of a comparative case-control study between posterior spinal arthrodesis and traditional growth rods in patients with juvenile idiopathic scoliosis, with an average age of 10.1 years for TGR and 10.8 years for patients with posterior spinal arthrodesis (AVP) [19]. Curvature correction at last follow-up was 63% in the AVP group and 44% in the TGR group. The mean increase in T1-S1 distance was 19% in the AVP group and 23% in the TGR group. The number of surgeries was significantly higher in the TGR group than in the AVP group. This research shows that there may be no benefit to TGR treatment in relatively older children with juvenile idiopathic scoliosis.

The advantage of using this unidirectional, self-expanding growth domino is that it reduces the number of surgeries, which has a major impact not only economically but also psycho-socially for families. Gaume et al. have demonstrated the absence of conversion to posterior vertebral arthrodesis after OWSER insertion [20]. One theory is that there may be an evolution toward ankylosis of the construct, without the need for final fusion. An extension of the study will show whether other cases require final arthrodesis or whether the ankylosis of the construct allows definitive maintenance of curvatures.

The main strength of our study is the prospective nature of patient follow-up. This is a study of an innovative, domestically developed device with few patients operated on in France. Our study is also comparable with the literature. Indeed, Gaume et al. found, in 21 patients, over a 36-month period, with an average age of 10.5 years, an average lengthening of the T1-T12 height of 60 mm and of the T1-S1 height of 80 mm, with an average decrease in Cobb angle of 34°. Thirty-eight percent (38%) of patients presented a complication.

The main limitations of this study are the small sample size and the difficulty of regular follow-up of these children with multiple comorbidities, resulting in missing data. In addition, the difficulty of sitting in the X-ray machines complicated the various angle and length measurements. Another limitation was the difficulty in collecting the answers to the questionnaire; indeed, a significant number of patients weren’t able to speak or write, which moderated our EOSQ-24 results. A subgroup analysis according to the different etiologies would provide a better understanding of the failures and enable us to target the etiologies most at risk.

## Conclusions

In these 20 patients with scoliosis, the use of OWSER resulted in significant lengthening of the spine and acceptable maintenance of curvature. The number of complications remains high but is comparable to the rest of the techniques described in the literature. We also highlight the fact that the device has a learning curve to be used properly by the surgeon, and this can also explain the complication rate of this study. We acknowledge the fact that this study is on a relatively small sample to draw definitive conclusions and that a longer follow-up period is required to confirm these results.
